# Organophosphate Pesticide Exposure and Breast Cancer Risk: A Rapid Review of Human, Animal, and Cell-Based Studies

**DOI:** 10.3390/ijerph17145030

**Published:** 2020-07-13

**Authors:** Kailynn June Yang, Jennifer Lee, Hannah Lui Park

**Affiliations:** Department of Epidemiology, School of Medicine, University of California, Irvine, CA 92697, USA; kailynny@uci.edu (K.J.Y.); jenniferlee815@gmail.com (J.L.)

**Keywords:** breast cancer, organophosphates, pesticides, carcinogenicity, endocrine disruption, anticholinesterase inhibition, mammary cancer

## Abstract

Background: Organophosphate pesticides (OPs) are one of the most commonly used classes of insecticides in the U.S., and metabolites of OPs have been detected in the urine of >75% of the U.S. population. While studies have shown that OP exposure is associated with risk of neurological diseases and some cancers, the relationship between OP exposure and breast cancer risk is not well understood. Methods: The aim of this rapid review was to systematically evaluate published literature on the relationship between OP exposure and breast cancer risk, including both epidemiologic and laboratory studies. Twenty-seven full-text articles were reviewed by searching on Pubmed, EMBASE, and Cochrane databases. Results: Some human studies showed that malathion, terbufos, and chlorpyrifos were positively associated with human breast cancer risk, and some laboratory studies demonstrated that malathion and chlorpyrifos have estrogenic potential and other cancer-promoting properties. However, the human studies were limited in number, mostly included agricultural settings in several geographical areas in the U.S., and did not address cumulative exposure. Conclusions: Given the mixed results found in both human and laboratory studies, more research is needed to further examine the relationship between OP exposure and breast cancer risk, especially in humans in non-agricultural settings.

## 1. Introduction

Breast cancer is the most common type of cancer among women worldwide after skin cancer, and approximately one in eight women will be diagnosed with invasive breast cancer in their lifetime [1]. Studies have suggested that only 5–10% of breast cancer cases are hereditary [2,3]. Thus, lifestyle and environmental factors have a significant impact on breast cancer risk. While factors such as obesity, physical activity, and alcohol consumption are known to be associated with breast cancer risk [4,5,6], the potential roles of environmental pollutants in breast cancer development are not well understood. However, a recent study comprised of 68,946 individuals found that a high consumption of organic foods was correlated with a decreased risk of postmenopausal breast cancer [7], suggesting that pesticide exposure may increase breast cancer risk.

Organophosphate pesticides (OPs) are currently one of the most commonly used classes of insecticides in the United States and are applied in agricultural, residential, and community settings. The global OP market is estimated to have a compound annual growth rate (CAGR) of 5.5% during the 2018–2023 period [8]. OPs present a societal, health, and environmental concern as they can poison not only insects but other animals as well, including birds, amphibians, and mammals. Exposure can occur through consumption of food and water containing OP residues, inhalation, or dermal absorption [9]. Outside of the agricultural setting, the primary source of OPs is through the diet [10]. According to the Environmental Protection Agency’s (EPA’s) Organophosphorus Cumulative Risk Assessment 2006 update, foods such as beans, watermelon, potatoes, and tomatoes are especially high in OPs [11]. According to data from the National Health and Nutrition Examination Survey (NHANES) from 2003 to 2004 of 2874 adults, OP exposure is prevalent; OP metabolites were detected in the urine of >75% of the U.S. population [12].

OPs are classified as anticholinesterases and thus increase the concentration of acetylcholine (ACh) within the neuromuscular junction. With high doses, this can lead to acute neurotoxicity in humans [13]. In the past decade, two types of OPs, parathion and diazinon, have been discontinued and banned for residential use, respectively [9,14]. In 2017, a petition was filed for the EPA to cancel all chlorpyrifos registrations, and in 2018 an order was issued by the U.S. Ninth Circuit Court of Appeals for the EPA to ban chlorpyrifos; however, this order was vacated in 2019 [15]. Thus, chlorpyrifos remains in use, including in agricultural, non-agricultural, and residential settings.

Several studies have demonstrated that OPs are also associated with cancer risk. For example, the Agricultural Health Study demonstrated that exposure to two other classes of OPs, malathion and terbufos, was significantly associated with risk for aggressive prostate cancer [16]. A recent meta-analysis also indicated a statistically significant association between OP exposure and non-Hodgkin’s lymphoma (NHL) risk [17]. In 2015, the International Agency for Research on Cancer (IARC) of the World Health Organization (WHO) classified malathion as “probably carcinogenic to humans” [18]. It has also been shown that some OPs possess endocrine-disrupting potential that may play a role in breast cancer development [19,20,21]. Given the widespread exposure to OPs and the high number of breast cancer cases, it is important to examine the relationship between OP exposure and breast cancer risk. While studies on OP exposure and human breast cancer risk have been limited, there have been numerous studies demonstrating the effect of OP exposure on mammary carcinogenesis in both animal and cell models. The purpose of this paper is to provide a rapid review of the current literature on both human and laboratory studies involving OPs and breast cancer.

## 2. Methods 

### 2.1. Study Design

This rapid review was designed and produced based on the PRISMA 2009 guidelines [22]. In light of the recently published NutriNet-Santé prospective cohort study that found a significantly decreased risk of breast cancer in postmenopausal women with high organic food consumption [7], a rapid review was conducted to provide a timely, comprehensive summary of the current literature surrounding the relationship between OPs and breast cancer risk, in the hopes of facilitating the conception of future studies on this topic. Given the variety of study methodologies, including human, animal, and cell-based studies, a quantitative meta-analysis of the quality of studies was not conducted.

### 2.2. Search Strategy and Inclusion/Exclusion Criteria

**Figure 1:**
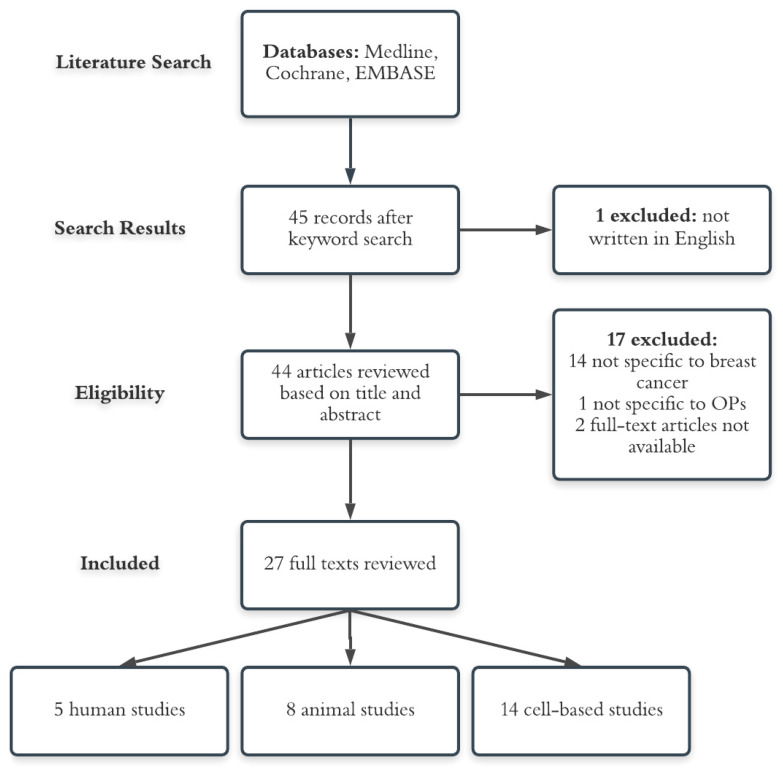
Flow chart summarizing study identification and selection process.

Pubmed, EMBASE, and Cochrane databases were used to conduct a systematic search based on the PRISMA guidelines (www.prisma-statement.org) for published literature on organophosphates and breast cancer development until 24 April 2020. The following search terms were used: (chlorpyrifos[Title/Abstract] OR acephate[Title/Abstract] OR malathion[Title/Abstract] OR naled[Title/Abstract] OR phorate[Title/Abstract] OR dicrotophos[Title/Abstract] OR dimethoate[Title/Abstract] OR terbufos[Title/Abstract] OR phosmet[Title/Abstract] OR ethoprophos[Title/Abstract] AND (breast[Title/Abstract] OR mammary[Title/Abstract]). These OP search terms were selected because they were listed as the top 10 most commonly used OPs in 2012 according to the U.S. EPA Pesticide Industry Sales and Usage 2008–2012 report [23], and are currently considered the most widely used OPs according to the 2019 Mordor Intelligence Global Organophosphate Pesticide Market report [8]. This search in PubMed resulted in 45 papers. No additional papers were identified by conducting the same search in Cochrane and EMBASE databases. One was excluded because it was not in English. Upon reading through the remaining 44 abstracts, two authors (KJY and HLP) agreed on the inclusion of 27 papers in this review. Studies were included if they were peer reviewed, written in English, and: (i) reported relative ratio (RR), hazard ratio (HR), or odds ratio (OR) with 95% confidence intervals (CI) on the association between the OP(s) and breast cancer risk in humans; or (ii) examined potential relationships between OP(s) and breast cancer development using animal or cell culture models. A flow chart summarizing the selection process is shown in Figure 1. 

### 2.3. Data Extraction

The search results are shown in Figure 1. Out of 45 search results, 27 full-text articles were included for review. All studies provided statistical analyses on the association between OPs and breast cancer risk or examined the potential relationship between individual OPs and characteristics of mammary carcinogenesis. Two researchers (KJY and HLP) independently extracted the following information, if available, from each human study: type of OP(s) analyzed; first author’s last name and publication year; study design, setting, population, enrollment period, follow-up period, and mean follow-up duration; exposure category/level; exposed cases/deaths; fully adjusted OR, HR, or RR and 95% CI; and strengths and weaknesses of the study. Extracted data for animal and cell-based studies were type of OP(s) analyzed; first author’s last name and publication year; cell line(s)/animal model(s) studied; dose(s) and duration of treatment; and outcome measures, summary of main findings, and potential mechanisms suggested in the paper.

### 2.4. Outcome Measures

The primary outcome measure for the human studies was breast cancer risk. The primary outcome measures for the animal studies were mammary tumorigenesis or measures of mammary gland carcinogenic potential, for example, counts of proliferating ducts, lobules, and/or terminal end buds. Other outcome measures included mean latency period; steroid hormone receptor expression; tumor growth rate; immunohistological and biochemical markers; and circulating hormone, DNA methylation, and histone deacetylase (HDAC) levels. The primary outcomes for the cell-based studies were measures or characteristics of mammary cell carcinogenic potential, for example, cell viability, anchorage independent growth, and cell invasion capabilities. Other outcome measures included levels of microsatellite instability (MSI) and loss of heterozygosity (LOH), estrogen receptor (ER) transactivation, changes in gene expression, reactive oxygen species (ROS) and reactive nitrogen species (RNS) production, and binding of OPs to sex hormones.

## 3. Results

### 3.1. Human Studies 

Our search results identified only five human studies. In 2005, a study compared women diagnosed with breast cancer (cases) to healthy women (non-cases) among the United Farm Workers (UFW) in the state of California [24]. Cases were identified using data from the California Cancer Registry in two time periods, 1988–1994 and 1995–2001, and exposure to OPs was estimated based on work histories supplied to the UFW linked to data from the California Department of Pesticide Regulation (CDPR) Pesticide Use Reports (PURs). The authors found that medium use of malathion by farm workers during 1988–1994, quantified in terms of pounds of malathion applied to a crop in a given county, month, and year, was significantly associated with increased breast cancer risk; however, there was no statistically significant difference in breast cancer risk for cases diagnosed during 1995–2001, which may be explained by differing patterns of usage within the two time periods or by chance due to the relatively small sample size.

Three studies were conducted using data from the Agricultural Health Study (AHS) in the states of Iowa and North Carolina. One of the primary goals of the AHS was to study the relationship between OP exposure and cancer risk among farmers and their spouses. In 2005, Engel and colleagues examined the association between OP exposure and breast cancer risk in spouses of pesticide applicators using data collected from an enrollment period between 1993–1997 and a follow-up period until 2000 [25]. Chlorpyrifos and terbufos were found to be marginally significantly and significantly, respectively, associated with increased breast cancer risk in women who were premenopausal at diagnosis. No other statistically significant associations were found. In 2015, a separate analysis found that any OP use and chlorpyrifos significantly and marginally significantly, respectively, increased breast cancer risk [26]. Any OP use also marginally significantly increased risk among postmenopausal women. In 2017, a follow-up of Engel et al.’s (2005) study examined the relationship between OP use at study enrollment and breast cancer risk with an average 14.7-year follow-up period, as well as OP use at enrollment in addition to OP use at the five-year follow-up period and breast cancer risk. They found that ever use of chlorpyrifos and terbufos non-significantly elevated breast cancer risk. In addition, malathion use at both enrollment and five-year follow-up significantly increased breast cancer risk but not use of malathion only at enrollment [27]. A comprehensive summary of this information in addition to the strengths and weaknesses of the studies can be found in Table 1.

A more recent study in 2019 compared residential and occupational exposures to several pesticides, including the OPs chlorpyrifos and diazinon, between breast cancer cases and controls in three counties of California’s Central Valley with the highest reported pesticide use and agricultural density [28]. Cases were identified from confirmed breast cancer diagnoses from 2007 to 2008 in the Cancer Registry of Central California (CRCC). Historic pesticide exposure among subjects was assessed using a Geographical Information Systems (GIS)-based method. This method combined state-reported pesticide use data from California PURs, land use surveys from California’s Department of Water Resources (DWR) Division of Planning and Local Assistance, and geocoded addresses to provide estimates of ambient pesticide exposure per acre within 500 m of each residential or occupational location. The study found that breast cancer was three times as likely to occur among women exposed to chlorpyrifos at residences or workplaces (or both) compared with those not exposed in either setting. There was also a moderate association between diazinon exposure and breast cancer; however, this was null after adjusting for use of other pesticides. Additionally, this study found that over 40% of residents and workers in the three counties, among both cases and controls, were exposed to ambient levels of one or more of the pesticides studied.

### 3.2. Animal Studies 

Our search criteria resulted in eight animal studies analyzing the association between OP(s) and mammary carcinogenic potential. Malathion was shown to significantly increase the number of proliferative ducts [29], actively growing tumors [30], number of ducts and lobules [31], and density of terminal end buds (TEBs), and shown to significantly decrease the density of alveolar buds (ABs) [32]. Chlorpyrifos was shown to significantly increase cell proliferation, number of ducts and hyperplastic ducts, HDAC mRNA levels [33], and tumor incidence, and decrease the latency period of tumor formation [34].

Some studies also suggested that chlorpyrifos and malathion may possess endocrine-disrupting potential. Chlorpyrifos and malathion significantly and non-significantly, respectively, decreased serum estradiol and progesterone levels [33,35]. Chlorpyrifos was also shown to significantly decrease co-repressors of estrogen receptor activity expression and significantly increase progesterone receptor expression [33]. However, one study showed no difference in circulating estradiol levels in chlorpyrifos-treated groups [36].

Suggested mechanisms for increasing mammary carcinogenic potential included acetylcholinesterase inhibition, endocrine disruption, increased oxidative stress, and decreased apoptotic signaling. A comprehensive summary of the results is shown in Table 2.

### 3.3. Cell-Based Studies

Our search criteria resulted in 14 cell-based studies analyzing the effects of OP exposure on mammary gland carcinogenesis. Two studies found that malathion, with or without estrogen, increased genomic instability, cell invasive capabilities and anchorage independent growth and altered the expression of cell cycle proteins in MCF-10F cells [39,40]. An additional study also demonstrated malathion-induced changes in gene expression [41]. In one study, malathion was found to weakly induce estrogen activity [42]. However, another concluded that malathion did not display any estrogenic activity [43]. Three studies demonstrated the estrogenic potential of chlorpyrifos by binding to sex hormones [44] and acting as estrogen agonists [45,46]. However, two other studies did not find any chlorpyrifos-induced endocrine disrupting potential [47,48]. Chlorpyrifos was also found to alter changes in gene expression [46], induce cell cycle arrest in the S and G2/M-phase vent [49], decrease cell proliferation [50], and increase oxidative stress [51]. A detailed summary of the main findings and their proposed cellular mechanisms are shown in Table 3.

## 4. Discussion

There has been recent concern over the use of pesticides, including OPs, and their implications on public health [52,53,54,55,56,57]. For example, several types of insecticides, fungicides, and herbicides have been found to be positively associated with breast cancer risk [58,59,60,61]. While the IARC classified malathion as “probably carcinogenic to humans” [18] and a recent meta-analysis indicated a statistically significant association between OP exposure and non-Hodgkin’s lymphoma (NHL) risk [17], a comprehensive review of studies examining the relationships between OPs and breast cancer risk had not been done. The present review highlights the existing literature on human, animal, and cell-based studies examining the relationships between OPs that are currently being used and breast cancer risk. 

Some associations were found in human studies linking OP exposure to increased breast cancer risk. While most results indicate a trend towards increased risk, only some were statistically significant. One study found a significantly increased risk of breast cancer associated with malathion [24], while another found a significantly increased risk of postmenopausal breast cancer associated with ever use of any OP [26]. Terbufos was also associated with a significantly increased risk of breast cancer among premenopausal women [27]. Likewise, chlorpyrifos was marginally significantly associated with increased breast cancer risk among premenopausal women, a finding that was consistent between two analyses within the Agricultural Health Study, one with an average of 4.8 years of follow-up and the other with an average of 14.7 years [25,27]. One study also indicated a marginally significant increased risk from chlorpyrifos in the unstratified group, but not specifically in premenopausal women [26]. Lastly, the central California Valley study found that residents and workers exposed to chlorpyrifos had a significantly higher risk of breast cancer than those not exposed [28]. The relatively consistent finding of chlorpyrifos being associated with increased breast cancer risk suggests that chlorpyrifos likely increases breast cancer risk, potentially more so in premenopausal women. It is interesting that, in two of the human studies [24,28], higher levels of OP use were associated with a lower OR than low exposure categories; however, the potential reasons for this were not addressed in the studies.

Numerous animal and cell-based studies linked malathion and chlorpyrifos, and one study linked dimethoate, to mammary carcinogenesis. The results suggest that these OPs can potentially increase breast cancer risk through various mechanisms including increased oxidative stress, disruption of adhesion molecules, acetylcholinesterase inhibition, endocrine disruption, and induction of genomic instability. However, while treatment with malathion resulted in increased cell proliferation, treatment of chlorpyrifos resulted in either increased cell proliferation or cell cycle arrest and apoptosis, perhaps depending on the dosage administered.

Since breast development is controlled by the endocrine system, endocrine disruption, defined as any disruption in the normal activity of the endocrine system [62], can lead to altered breast development and may increase breast cancer risk. Studies suggest that chlorpyrifos and malathion may possess such endocrine disrupting potential, but the results were not consistent. Most of the studies suggested that chlorpyrifos and malathion are potential estrogen agonists and thus may activate the ERa and AhR pathway, leading to cellular proliferation and other mammary cell disruptions. Among the in vivo studies, one reported increased cellular and lobular proliferation along with decreased estradiol and progesterone levels among chlorpyrifos-treated rats [33], while another reported decreased steroid hormone receptor expression [34]. However, one found no difference in circulating hormone levels among chlorpyrifos-treated rats compared to control rats [36], suggesting that chlorpyrifos does not possess endocrine-disrupting potential. Among the in vitro studies, one reported that malathion and dimethoate did not possess any endocrine disrupting activity while another reported a mild endocrine disrupting activity through activation of the ERa pathway in malathion-treated breast cancer cells [42,43]. Chlorpyrifos was also shown to act as a potential Era and AhR agonist among two studies thereby increasing cell proliferation and viability that may eventually lead to breast tumorigenesis [46,49]. However, chlorpyrifos did not induce cell proliferation or exhibit ER activation in two other studies [47,48] and was found to decrease cell proliferation through mechanisms that may or may not involve the ER pathway [45,50,51], demonstrating the potential cytotoxic effects chlorpyrifos has on breast cell lines through endocrine disrupting mechanisms. Chlorpyrifos also demonstrated binding affinity to sex hormones estradiol and estrone, further demonstrating its endocrine disrupting potential [44]. 

**Figure 2:**
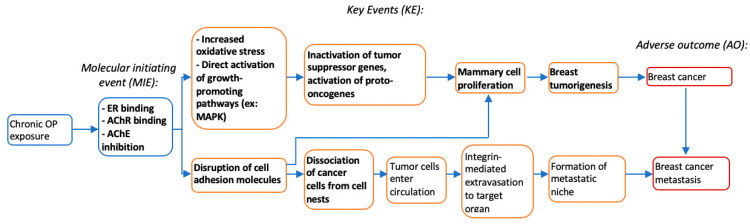
Adverse outcomes pathway including proposed mechanisms of chronic OP exposure that may lead to mammary carcinogenesis. Events shown in bold are mechanisms observed or proposed by the studies in this review [63,64,65,66,67,68,69,70,71,72].

Studies discussed in this review also observed increased cholinergic activity in OP-treated breast cancer cells and animal models [31,32,39]. Whether this can be attributed to higher ACh levels due to the anticholinesterase effects of OPs or to the OPs acting directly on acetylcholine receptors (AChRs) as agonists is unclear. Of the two types of AChRs, muscarinic (mAChR) and nicotinic (nAChR), activation of the M3 mAChR subtype has been shown to induce proliferation in human breast cells, as well as colon and prostate cells, potentially through activation of the MAPK/ERK pathway [63]. nAChRs have also been shown to play a role in cell proliferation, tumor invasion, and angiogenesis [64]. In breast cancer cells specifically, it has been shown that exposure to nicotine increases expression of the alpha-9 subtype of nAChR (a9-nAChR), which in turn promotes cell proliferation and colony formation in the MCF-10A cell line [65]. With anticholinesterase activity being one of the more widely recognized effects of organophosphates, it may play a significant role in mammary carcinogenesis. An adverse outcomes pathway based on these findings is presented in Figure 2. 

There were several limitations among the current literature. Among the human studies, two had a case control study design [24,28]; thus, they are subject to selection bias. In addition, one of the cohort studies only had a mean 4.8 years of follow-up [25]. All but one was done in an agricultural setting, limiting the generalizability of the results. The one human study that was done in a non-agricultural setting relied on a GIS method that combined land use data with historic pesticide application within a certain radius of households and workplaces. While the GIS method can be viewed as a strength due to the absence of self-reporting bias, and despite GIS being increasingly used in population-based studies, there are some limitations of GIS worth noting [28]. First, publicly available data are generally reserved for legislative purposes, unrelated to healthcare. Second, in order for GIS extrapolations to be accurate, data need to be complete and accurate, which georegistered data often are not [73]. While this study did control for several GIS limitations, such as migration, inferences drawn should be taken with caution given these inherent issues of GIS.

Mills and colleagues conducted an electronic record linkage recording ever use of OPs among members of the UFWA, which eliminated the issue of recall bias but introduced other limitations [24]. By using an ecological assessment method, which involved classifying pesticide exposure based on the location (county), crop, and year involved, the UFWA study was able to quantify pesticide use into high vs. medium vs. low use, but failed to assess individual exposure to pesticides and exposure due to non-agricultural sources, such as in the home, which could have also led to exposure misclassification. There is also the possibility of an undercount of breast cancer cases in the UFWA Mills study due to the lack of healthcare access among some women.

Among all the studies, due to the nature of the collection method for exposure assessment, lifetime cumulative use of individual insecticides was not assessed. Studies involving the AHS cohort only collected information from a self-administered questionnaire about ever/never use during the pre-enrollment period [25,26] and five-year follow-up [27]. Thus, limited information about duration or time-period of use for individual pesticides was obtained, which may have led to exposure misclassification. This can also mean limited information regarding each pesticide’s application, mode, concentration, quantity, frequency, and potential combination with another pesticide. Additionally, the timing of exposure may be a significant factor to consider as some pesticides can mimic estrogen, thus potentially affecting mammary cells especially during periods of mammary gland growth and differentiation, such as during pregnancy. Moreover, self-reported questionnaire data is prone to reporting bias and/or inaccurate recall of information.

There are also likely some discrepancies in the true OP levels individuals were exposed to, as exposure can occur directly, through applying or mixing pesticides, or indirectly, through handling items, food, or water containing OP residues or through aerial drift. Exposure is also dependent on the type of protective equipment used during application and handling, as well as the pathway of exposure. For example, exposure can occur through dermal, inhalation, or ingestion routes [74], dermal being the most applicable to agricultural workers [75]. In addition, the UFWA study had a small sample size while studies involving the AHS cohort only had a small number of cases exposed to certain OPs, leaving the possibility of some of the associations due to chance. Furthermore, since many of the women were likely exposed to more than one pesticide with similar mechanisms of action, some associations may have been overestimated.

Lastly, none of the human studies used biomonitoring collection methods as a way of measuring OP exposure. Environmental exposure to OPs occurs primarily via ingestion, inhalation, and dermal contact [76,77,78]. After absorption, OPs make their way into the blood circulatory system, are metabolized by Phase I and Phase II enzymes, and then excreted in the urine [77,78]. Biomonitoring methods for OPs usually aim to detect their metabolites, which most commonly include dialkyl phosphates (DAPs), in the blood, urine, saliva, or tissue. DAP metabolites include dimethylphosphate (DMP), dimethylthiophosphate (DMTP), dimethyldithiophosphate (DMDTP), diethylphosphate (DEP), diethylthiophosphate (DETP), and diethyldithiophosphate (DEDTP), which are present even after low levels of OP exposure [76]. Due to the fast, metabolic nature of OPs and their high excretion rates, biomonitoring of the urine often only reflects recent exposure to OPs. However, urinary elimination may reach a steady state with non-fluctuating metabolic levels, representing a chronic exposure to OPs [79]. While biomonitoring provides a rough estimate, it is often considered a more biologically accurate way of measuring OP exposure. Future studies should consider using the highly specific method of gas chromatography coupled with tandem mass spectrometry for biomonitoring [80,81,82].

There were also some limitations among the animal and cell-based studies. An inherent limitation of cell culture studies is that the pesticides would not be metabolized and cleared in the same manner as they would in a human. In addition, there is some evidence that cell lines show less sensitivity to their extracellular environment compared to cell cultures from tissue samples [83]; thus, the use of cell lines may attenuate the true associations between OPs and characteristics of mammary carcinogenesis. In addition, most of the in vitro studies were done using breast cancer cell lines, which may respond differently from non-cancerous mammary cells, which would be more a more appropriate model system to study potential risk factors [84]. The inconsistencies between some of the studies can likely be explained by differences in treatment methodologies, assays, endpoints, cell lines, and rodent populations. For example, in one study, malathion and dimethoate did not exhibit endocrine disrupting potential while in another, malathion exhibited weak estrogenic potential [43,44]. The former administered increasing concentrations of malathion between 10^−11^ and 10^−6^ M, which represents the concentration range deemed safe by the National Institute of Environmental Research (NIER), while the latter administered a concentration of 10^−5^ M of malathion, which could potentially explain the discrepancies. Thus, some experimental doses of OPs may have limited applicability to real-life environmental exposure levels.

Another limitation shared by both human and animal studies in this review is the lack of survival or mortality data. The animal studies’ measurement outcomes were limited to breast tumor growth and tissue histological changes and did not continue on to observe mammary cancer development, metastasis, or cancer-related death. In human studies, breast cancer cases were generally limited to surviving and lower-stage cancers. Future studies should incorporate longer follow-up and include mortality analyses.

In this rapid review, we examined the current literature on human, animal, and cell-based studies that analyzed the potential association between OP exposure and breast cancer risk. To our knowledge, this is the first review on the relationship between OPs and breast cancer risk. One limitation on the review level is that the analyses may lack compatibility due to differences in study populations, cell lines, data collection methods, treatment methodologies, and follow-up times, making it difficult to compare the results among studies with similar endpoints. However, the advantage of such a review is the increased sample size and the ability to compare data and potential confounders among a diverse set of results, allowing us to draw novel inferences from many sources combined. Due to the nature of the study being a rapid review, another limitation is the absence of a quality assessment. Publication bias, the bias towards reporting positive associations, is also a possibility.

## 5. Conclusions

The findings of this rapid review on human, animal, and cell-based studies suggest that certain OPs may be involved in increasing breast cancer risk. The results from the human studies demonstrated that malathion, chlorpyrifos, terbufos, and phorate may be associated with an increased risk of breast cancer. In addition to acting as acetylcholinesterase inhibitors, evidence suggests that malathion and chlorpyrifos have the potential to cause endocrine disruption, induce oxidative stress, and alter the expression of cell cycle proteins and cell adhesion molecules important for breast development. Taken together, given the prevalence of OPs and number of breast cancer cases, there is a need for further human research in non-occupational settings, potentially including the assessment of cumulative lifetime exposure to OPs, biomonitoring, mortality data, and a wider geographical assessment.

## Figures and Tables

**Table 1:** Summary of results, strengths, and weaknesses for human studies on organophosphate pesticides and breast cancer risk.

First Author, Year Published	Study Design, Setting, Population, and Period	Exposure Assessment	OP	Exposure Category/ Level (Cases vs. Controls)	Results	Strengths	Weaknesses
Mills, 2005 [24]	Case control, Hispanic agricultural workers in California; Cases diagnosed 1988–1994	Union work histories were linked to the Department of Pesticide Regulation (DPR) Pesticide-Use Reports (PURs) for relevant crop within a given month/year in a given county	Malathion	Low use (14 cases, 62 controls)	OR = 1.89 (95% CI: 0.72, 4.94)	Data based on registry information, so no reporting or selection bias. Assessed differential (high vs. medium vs. low) use of pesticides.	Possible undercount of breast cancer cases due to limited healthcare access by some UFW members. Relatively small sample size. No information on job tasks or protective clothing could have led to exposure misclassification. Unable to assess lifetime cumulative use. Did not analyze exposure from other sources.
				Medium use (16 cases, 52 controls)	**OR = 2.95** (95% CI: 1.07, 8.11)		
				High use (9 cases, 60 controls)	OR = 1.68 (95% CI: 0.50, 5.62)		
			Diazinon	Low use (9 cases, 53 controls)	OR = 0.78 (95% CI: 0.12, 4.48)		
				Medium use (17 cases, 68 controls)	OR = 1.54 (95% CI: 0.22, 10.68)		
				High use (10 cases, 58 controls)	OR = 1.50 (95% CI: 0.18, 12.35)		
	Case control, Hispanic agricultural workers in California; Cases diagnosed 1995–2001		Malathion	Low use (17 cases, 85 controls)	OR = 0.79 (95% CI: 0.40, 1.56)		
				Medium use (18 cases, 95 controls)	OR = 0.68 (95% CI: 0.33, 1.43)		
				High use (14 cases, 87 controls)	OR = 0.50 (95% CI: 0.21, 1.23)		
			Diazinon	Low use (20 cases, 96 controls)	OR = 1.18 (95% CI: 0.27, 5.20)		
				Medium use (21 cases, 8 controls)	OR = 1.42 (95% CI: 0.30, 6.81)		
				High use (13 cases, 91 controls)	OR = 0.76 (95% CI: 0.15, 3.92)		
Engel, 2005 [25]	Cohort, spouses of pesticide applicators in Iowa and North Carolina; Enrollment period: 1993–1997 Follow-up period: 1993–2000 (mean 4.8 years)	Questionnaire; direct and indirect use at enrollment (n = 30,594)	Any OP use	Ever/never use (86 exposed cases, 7580 exposed controls)	RR = 1.0 (95% CI: 0.8, 1.3)	Prospective, longitudinal design, large sample size, comprehensive exposure assessment, extent of potential confounder control, and exploration of potential effect modulation, such as by family history. Little or no loss to follow-up.	Relatively short follow-up duration and the modest number of cases limited interpretation of the results. Number of exposed cases of premenopausal women was small. Few cases exposed to less commonly used pesticides. Unable to assess lifetime cumulative use. No data about the length of marriage; limited information about the extent of indirect exposure via husband’s use. Self-reported questionnaires may have introduced some reporting bias or inaccurate recall.
				Premenopausal	RR = 1.1 (95% CI: 0.7, 1.7)		
				Postmenopausal	RR = 0.9 (95% CI: 0.7, 1.3)		
			Malathion	Ever/never use (63 exposed cases, 5706 exposed controls)	RR = 0.9 (95% CI: 0.7, 1.2)		
				Premenopausal	RR = 0.9 (95% CI: 0.5, 1.5)		
				Postmenopausal	RR = 0.9 (95% CI: 0.6, 1.2)		
			Diazinon	Ever/never use (31 exposed cases, 2977 exposed controls)	RR = 1.0 (95% CI: 0.7, 1.5)		
				Premenopausal	RR = 0.8 (95% CI: 0.4, 1.6)		
				Postmenopausal	RR = 1.1 (95% CI: 0.7, 1.8)		
			Chlorpyrifos	Ever/never use (16 exposed cases, 1162 exposed controls)	RR = 1.4 (95% CI: 0.9, 2.4)		
				Premenopausal	**RR = 2.2** (95% CI: 1.0, 4.9)		
				Postmenopausal	RR = 1.0 (95% CI: 0.5, 2.2)		
			Terbufos	Ever/never use (10 exposed cases, 838 exposed controls)	RR = 1.1 (95% CI: 0.6, 2.1)		
				Premenopausal	**RR = 2.6** (95% CI: 1.1, 5.9)		
				Postmenopausal	RR = 0.5 (95% CI: 0.2, 1.6)		
			Phorate	Ever/never use (6 exposed cases, 575 exposed controls)	RR = 0.8 (95% CI: 0.4, 1.8)		
				Premenopausal	RR = 1.5 (95% CI: 0.5, 4.9)		
				Postmenopausal	RR = 0.6 (95% CI: 0.2, 2.0)		
Lerro, 2015 [26]	Nested case case, spouses of pesticide applicators, Iowa and North Carolina; Enrollment period: 1993–1997 Follow-up period: 1993–2011 (mean 15.3 years)	Questionnaire; direct and indirect use at enrollment (n = 29,325)	Any OP use	Ever/never use (296 exposed cases, 763 non-exposed cases)	**RR = 1.20** (95% CI: 1.01, 1.43)	Longitudinal design with regular linkage to population registries for cancer and mortality outcomes and little or no loss-to-follow-up. Large sample size, comprehensive exposure assessment, extent of potential confounder control, and exploration of potential effect modulation, such as by family history.	Only ~25% of participants reported ever use at enrollment. Only collected information on lifetime ever use, no information on duration or time period of use. Did not distinguish between occupational versus non-occupational use. Self-reported questionnaires may have introduced some reporting bias or inaccurate recall. Did not evaluate indirect exposure.
				Premenopausal	RR = 1.02 (95% CI: 0.77, 1.36)		
				Postmenopausal	**RR = 1.27** (95% CI: 1.00, 1.62)		
			Malathion	Ever/never use (223 exposed cases, 836 non-exposed cases)	RR = 1.05 (95% CI: 0.88, 1.26)		
				Premenopausal	RR = 1.04 (95% CI: 0.78, 1.38)		
				Postmenopausal	RR = 1.03 (95% CI: 0.81, 1.30)		
			Chlorpyrifos	Ever/never use (50 exposed cases, 1009 non-exposed cases)	**RR = 1.41** (95% CI: 1.00, 1.99)		
				Premenopausal	RR = 1.36 (95% CI: 0.79, 2.34)		
				Postmenopausal	RR = 1.53 (95% CI: 0.96, 2.44)		
			Terbufos	Ever/never use (37 exposed cases, 1022 non-exposed cases)	RR = 1.52 (95% CI: 0.97, 2.36)		
				Premenopausal	RR = 1.25 (95% CI: 0.61, 2.54)		
				Postmenopausal	RR = 1.73 (95% CI: 0.93, 3.21)		
			Diazinon	Ever/never use (118 exposed cases, 941 non-exposed cases)	RR = 1.14 (95% CI: 0.93, 1.38)		
				Premenopausal	RR = 1.01 (95% CI: 0.73, 1.40)		
				Postmenopausal	RR = 1.11 (95% CI: 0.85, 1.45)		
Engel, 2017 [27]	Cohort study, spouses of pesticide applicators, Iowa & North Carolina; Enrollment period: 1993–1997 Follow-up period: 1993–2011 (mean 14.7 years)	Questionnaire, direct and indirect use at enrollment and at 5-y follow-up interview; ever vs. never use (n = 30,594)	Any OP use	Ever/never use (300 exposed cases, 7389 exposed controls)	HR = 1.1 (95% CI: 0.9, 1.2)	Large sample size, direct and indirect exposure assessment of many pesticides before breast cancer diagnosis, extent of potential confounder control, and exploration of potential effect modulation, such as by family history. Long follow-up period including detailed data.	Few women reported use at follow-up. No cumulative assessment available except for in post enrollment period. Limited exposed cases for some OPs. Unable to assess early lifetime exposure. Self-reported questionnaires may have introduced some reporting bias or inaccurate recall.
				Premenopausal	HR = 1.1 (95% CI: 0.8, 1.6)		
				Postmenopausal	HR = 1.0 (95% CI: 0.9, 1.2)		
			Chlorpyrifos	Ever/never use (51 exposed cases, 1130 exposed controls)	**HR = 1.4** (95% CI: 1.0, 2.0)		
				Premenopausal	**HR = 1.9** (95% CI: 1.0, 3.8)		
				Postmenopausal	HR = 1.3 (95% CI: 0.9, 1.9)		
			Diazinon	Ever/never use (118 exposed cases, 2,902 exposed controls)	HR = 1.1 (95% CI: 0.9, 1.3)		
				Premenopausal	HR = 1.1 (95% CI: 0.6, 1.8)		
				Postmenopausal	HR = 1.1 (95% CI: 0.8, 1.3)		
			Malathion	Ever/never use (226 exposed cases, 5561 exposed controls)	HR = 1.0 (95% CI: 0.8, 1.2)		
				Premenopausal	HR = 0.9 (95% CI: 0.6, 1.4)		
				Postmenopausal	HR = 1.0 (95% CI: 0.8, 1.2)		
			Phorate	Ever/never use (22 exposed cases, 561 exposed controls)	HR = 1.1 (95% CI: 0.7, 1.8)		
				Premenopausal	**HR = 2.5** (95% CI: 1.0, 6.2)		
				Postmenopausal	HR = 0.9 (95% CI: 0.5, 1.6)		
			Terbufos	Ever/never use (37 exposed cases, 814 exposed controls)	**HR = 1.5** (95% CI: 1.0, 2.1)		
				Premenopausal	**HR = 2.6 (95% CI: 1.3, 5.4)**		
				Postmenopausal	HR = 1.2 (95% CI: 0.8, 1.9)		
Tayour, 2019 [28]	Case control, Non-Hispanic white residents or workers in the California Central Valley	Geographical Information System (GIS)-based method which combines California PUR data, California Department of Water Resources (DWR) land use surveys, and geocoded addresses with ambient pesticide exposure within 500 m of residences or workplaces	Chlorpyrifos	Exposed at residences only (17 cases, 9 controls)	**OR = 3.91** (95% CI: 1.29, 11.85)	Use of address histories and registry data reduce potential of recall bias and exposure misclassification. Controlled for known breast cancer risk factors.	Smaller study (155 cases, 150 controls) without dose-response data. Breast cancer cases participating in study were limited to surviving cases and lower stage breast cancers.
				Exposed at workplaces only (21 cases, 18 controls)	**OR = 2.90** (95% CI: 1.12, 7.54)		
				Exposed at both residences and workplaces (82 cases, 64 controls)	**OR = 2.27** (95% CI: 1.18, 4.38)		
			Diazinon	Exposed at residences only (8 cases, 9 controls)	OR = 1.06 (95% CI: 0.32, 3.50)		
				Exposed at workplaces only (18 cases, 17 controls)	OR = 1.23 (95% CI: 0.47, 3.26)		
				Exposed at both residences and workplaces (92 cases, 79 controls)	OR 1.30 (95% CI: 0.69, 2.45)		

Significant and marginally significant associations are bolded. OP, organophosphate pesticide; UFW, United Farm Workers.

**Table 2:** Summary of animal studies on organophosphate pesticides and mammary gland carcinogenesis.

OP	First Author, Year Published	Animal Model/ Strain	Treatment	Outcome Measures	Main Findings	Suggested Mechanism(s)	Additional Notes
Chlorpyrifos	Kang, 2004 [36]	20-day-old Sprague-Dawley rats	Injected sc with 2, 10, 50, or 250 mg/kg chlorpyrifos, 2 μg/kg 17ß-estradiol per day for 3 days	Circulating hormone levels	No difference in circulating estradiol levels in group treated with chlorpyrifos compared to group treated with vehicle alone.	Chlorpyrifos can potentially act as an endocrine disruptor.	
Chlorpyrifos	Ventura, 2016 [33]	40-day-old virgin female Sprague-Dawley rats	Ingested orally 0.01 or 1 mg/kg/day for 100 days	Cholinesterase activity; number of ducts and lobular buds; percent of hyperplastic ducts and lobular adenosis; cell proliferation; PCNA staining; PgR and ERa expression; co-repressor of estrogen receptor activity expression; circulating hormone levels	AChE activity not affected; **decreased BChE activity; increased number of ducts in 0.01/mg/kg/day treatment group** compared to controls treated with vehicle alone; no change in number of lobular buds; **increased percentage of hyperplastic ducts in 1/mg/kg/day** and 0.01/mg/kg/day treatment groups; **increased percentage of lobular adenosis in 0.01 mg/kg/day**; no changes observed for 1 mg/kg/day; **increased cell proliferation in 1/mg/kg/day and 0.01/mg/kg/day** treatment groups; **increased PgR expression at both doses**; no changes in ERa expression; **decreased co-repressors of estrogen receptor activity for 0.1 mg/kg/day treatment group; decreased serum estradiol and progesterone for 1 mg/kg/day treatment group; decreased serum LH at both doses**; no change in FSH; 1 mg/kg/day prevented an LH increase after ovariectomy.	Chlorpyrifos is a potential endocrine disruptor and may induce cellular proliferation and other mammary cell disruptions.	0.01 mg/kg/day is the Acceptable Daily Intake level; 1 mg/kg/day is the No Observed Adverse Effects level.
Chlorpyrifos	Ventura, 2019 [34]	40-day-old virgin female Sprague-Dawley	Ingested orally 0.1 or 1 mg/kg/day + 50 mg/kg body weight NMU at 50, 80, and 110 days old	Mammary tumor incidence; tumor doubling time; latency period, number of tumors per rat; steroid hormone receptor expression levels; DNA methylation and HDAC levels	**Increased tumor incidence in both 0.01 and 1 mg/kg/day treatment groups at 110 days** but no difference at 150 days; **reduced latency period in tumor formation at both doses**; increased number of tumors per rat in both treatment groups; no effect on tumor doubling time; decreased ERa and PgR expression; no effect on CDKN1B and BRCA1 promoter methylation levels; **increased HDAC mRNA levels in 0.1 mg/kg/day treatment group**.	Chlorpyrifos is a potential endocrine disruptor and may alter mammary gland structures.	
Malathion	Silinskas, 1975 [30]	36-day-old female Sprague-Dawley rats	Treated with DMBA, a chemical carcinogen, for 230 days, with or without pre-treatment with 250 ppm malathion via ingestion for 14 days	Mammary gland tumorigenesis; mean latency period; number of tumors per rat; tumor growth	29/29 rats treated with malathion + DMBA developed mammary tumors, compared to 26/28 rats treated with DMBA alone; rats treated with malathion + DMBA had a shorter mean latency period and higher number of tumors per rat; **rats treated with malathion + DMBA had more actively growing tumors**.	Malathion inhibits steroid degradation via hydroxylation and may enhance hormone dependent DMBA action.	
Malathion	Cabello, 2001 [32]	16-day-old and 39-day-old, virgin female Sprague-Dawley rats	Injected sc with 17 mg/100 g bw malathion with or without 250 µg/100 g bw atropine, an anticholinergic drug, twice a day for five days	Mammary gland tumorigenesis; density of terminal end buds; density of alveolar buds	17/70 malathion-treated rats developed mammary tumors compared to 0/70 control saline-treated rats; 39-day-old malathion-treated rats had **increased density of TEBs and decreased density of ABs compared to control**; 39-day-old atropine + malathion-treated rats had **decreased density of TEBs and increased density of ABs** compared to malathion-treated rats; no difference in density of TEBs or ABs in 16-day-old treated rats compared to control.	Increased cholinergic stimulation alters enzymatic pathways controlling the cell cycle, promoting TEB proliferation and preventing differentiation into ABs, as seen in mammary carcinogenesis	Different age groups were treated since previous studies demonstrated that rats at different age groups treated with DMBA displayed significantly different results [37,38].
Malathion	Calaf, 2011 [29]	39-day-old virgin female Sprague-Dawley rats	Injected sc with malathion 22 mg/100 g bw, with or without 17ß-estradiol 30 µg/100 g bw, twice a day for 5 days	Number of proliferative ducts per mm^2^; average number of secretory lobules per mm^2^	**Increased average number of proliferative ducts per mm**^**2**^**in malathion-treated rats compared to control and rats treated with estrogen only; estrogen-treated rats had an increased number of secretory lobules compared to other groups**.	Malathion may act as an estrogen agonist, enhancing the effects on mammary structures when treated in combination with estrogen.	Specific peaks of catechol estrogens 2-CE and 4-CE in rats with mammary tumors indicated potential usefulness as mammary cancer biomarkers.
Malathion	Calaf, 2012 [31]	39-day-old virgin female Sprague-Dawley rats	Injected sc with malathion 22 mg/100 g bw, with or without 17ß-estradiol 30 µg/100 g bw, or with or without atropine 250 µg/100 g bw, twice a day for 5 days	Number of lobules; number of ducts; immunohistological markers	**Increased average number of ducts and lobules** in estrogen + malathion treated rats compared to control and atropine-treated rats; increased CYP1A1, mutant p53, c-myc, and c-fos expression; malathion + estrogen displayed synergism; atropine counter-acted effects of malathion.	AChE inhibition; oxidative stress.	
Malathion	Omran, 2015 [33], [35]	39-day-old female Wistar albino rats	Injected ip with 170 mg/kg bw malathion, with or without α-lipoic acid 20 mg/kg bw, twice a day for 5 days	Mammary gland tumorigenesis; immunohistological markers; biochemical markers	6/10 rats treated with malathion developed mammary ductal carcinomas compared to 0/10 rats in the control group and 1/10 rats treated with malathion and α-lipoic acid; decreased BAX, increased PCNA, and increased mutant p53 protein expression in malathion treated group; decreased FSH, estradiol and progesterone secretion; decreased catalase and superoxide mutase activity; **α-lipoic acid, an antioxidant, counteracted effects induced by malathion (as measured by catalase)**.	Decreased apoptotic signaling; free radical generation; inhibition of pituitary gonadotropin secretion.	

Findings that are bolded were statistically significant. DMBA, 7,12-Dimethylbenz[a]anthracene; TEB, terminal end buds; AB, alveolar buds; PCNA, proliferating cell nuclear antigen.

**Table 3:** Summary of in vitro studies on organophosphate pesticides and mammary cell carcinogenesis.

OP	First Author, Year Published	Cell Line(s)	Treatment	Outcome Measures	Main Findings	Proposed Mechanism(s)
Chlorpyrifos	Vinggaard, 1999 [47]	MCF-7	Treated with 0.001, 0.01, 1, and 10 uM chlorpyrifos for 6 days. For cell proliferation assays; 0.24–500 uM final concentration for 4 days	Cell proliferation; estrogen receptor activation	Chlorpyrifos did not induce cell proliferation or exhibit estrogen receptor activation.	
Chlorpyrifos	Rich, 2012 [45]	MCF-7, MDA-MB-231, and MCF-10A	Treated with 10, 100, 1000, and 10000 nM chlorpyrifos for 48 h	Cell viability	Decreased cell viability by 37% in MCF-7 lines treated with 10,000 nM but this was not statistically significant	May involve estrogen receptor.
Chlorpyrifos	Ventura, 2012 [49]	MCF-7 and MDA-MB-231	Treated with 0.05, 0.5, 5, or 50 uM chlorpyrifos for 10 days	Cell proliferation; ROS production; ERα transactivation; cell cycle protein distribution in G1-S and S phase	0.05 uM induced cell proliferation while 50 uM induced S-phase arrest in MCF-7; 50 uM induced G2/M phase arrest in MDA-MB-231	Induces cellular proliferation by acting as an estrogen agonist and activating the ERα pathway. Induces cell cycle arrest, possibly by directly altering MT polymerization or by altering redox balance.
Chlorpyrifos	Dellai, 2013 [50]	MCF-7	Treated with 0–200 µg/mL chlorpyrifos, with and without *P. peli* up to 4 days	Cell viability; cell proliferation	Decreased cell proliferation up to 50% in dose-dependent manner; this was attenuated by the addition of *P. peli*	*P. peli* metabolizes chlorpyrifos, reducing its cytotoxic effects.
Chlorpyrifos	Medjakovic, 2014 [48]	MCF-7 and MDA-MB-231	Treated with 10 uM and 100 uM chlorpyrifos for 24, 48, and 72 h	ERα transactivation; cell growth	Did not transactivate ERa; weakly inhibited cell growth	Can act as a potential estrogen agonist.
Chlorpyrifos	Farhadi, 2015 [44]	N/A	0.75 to 142.62 uM chlorpyrifos + either estrone, 17β-estradiol, and DES	Binding of chlorpyrifos to estrogens	Exhibited high-affinity specific binding for estrone and estradiol and an intermediate affinity binding site for DES under near physiological conditions	Chlorpyrifos may act as an endocrine disruptor by binding to sex hormones.
Chlorpyrifos	Ventura, 2015 [51]	MCF-7 and MDA-MB-231	Treated with 50 uM chlorpyrifos for different times, depending on assay	Cell proliferation; ROS and RNS production; catalase and SOD activity lipid peroxidation; ERK1/2 phosphorylation	Decreased cell proliferation; increased ROS production in both cell lines and increased RNS production in MDA-MB-231; increased catalase activity in both cell lines and decreased SOD activity in MCF-7; increased lipid peroxidation in MCF-7 cells; increased ERK1/2 phosphorylation in both lines.	Increases H2O2 levels, inducing phosphorylation of ERK1/2, which leads to inhibition of cellular proliferation and apoptosis.
Chlorpyrifos	Moyano, 2020 [46]	MCF-7 and MDA-MB-231	Treated with 0.01 to 100 uM chlorpyrifos and its derivative CPFO for 24 h and 14 days	cell proliferation; KIAA1363 enzyme, AhR, Era, and CYP1A1 expression	CPF and its derivative CPFO altered KIAA1363 enzyme, AhR, ERa and CYP1A1 expression, increased cell proliferation in both cell lines through ERa activation in MCF-7 cell lines after 24 h and through KIAA1363 overexpression and AhR activation in both cell lines at both 24 h and 14 days	In addition to acting as an ERa agonist, CPF can alter the expression of the KIAA1363 enzyme leading to cellular proliferation.
Dimethoate	Chen, 2002 [43]	MCF-7	Treated with a 10^−11^ to 10^−6^ M dimethoate and 10^−9^ M estradiol for 144 h	Cell proliferation; ER-competitive binding	Did not significantly increase cell proliferation or inhibit binding of estradiol	Can act as an estrogen agonist and induce mammary cell proliferation but did not display estrogenic activity.
Malathion	Chen, 2002 [43]	MCF-7	Treated with 10^−11^ to 10^−6^ M malathion and 10^−9^ M estradiol for 144 h	Cell proliferation; ER competitive binding	Did not significantly increase cell proliferation or inhibit binding of estradiol	Can act as an estrogen agonist by inducing cellular proliferation and inhibiting the binding of estradiol to the ER but did not display estrogenic activity.
Malathion	Gwinn, 2005 [41]	Normal human mammary epithelial cells from 4 strains in their 6th passage	Treated with mixture of 50 µL/mL malathion for 6 and 24 h	Cell viability; changes in gene expression	Did not significantly affect cell viability; increased expression of AKR1C1, AKR1C2, EBBP and decreased expression of PLAT, CPF, RFC3, TYMS, BUB1, and AI859865 in all four cell strains	Alters carcinogen and steroid metabolism, DNA replication, and cell cycle progression.
Malathion	Calaf, 2008 [39]	MCF-10F in their 44th passage	Treated for 20 passages with malathion (100 ng/mL) with or without 10^−8^ M estradiol	Anchorage independent growth; invasive capabilities; gene expression	Cells treated with malathion with or without estradiol had increased anchorage independent growth and invasive capabilities and increased expression of cyclins, CDK-4, IGFBP3, IGFBP5, keratin-18, c-Ha-ras, HSP 27, MCM2, and TP53	Increases cholinergic stimulation, altering gene expression of proteins important for cell cycle regulation.
Malathion	Calaf, 2009 [40]	MCF-10F in their 20th passage	Treated with malathion (2 µL/mL) with or without 10^−8^ M estradiol for 2 weeks	Anchorage independent growth; invasive capabilities; mutant p53 and c-Ha-ras protein expression; MSI and LOH	Cells treated with malathion with or without estradiol had increased anchorage independent growth and invasive capabilities, increased expression of mutant p53 and c-Ha-ras; increased MSI and LOH	Induces malignant transformation through genomic instability of p53 and c-Ha-ras.
Malathion	Kjeldsen, 2013 [42]	MVLN (MCF-7 derivative)	Treated with 10^−9^ to 10^−4^ M malathion for 18–24 h	ER transactivation	Treatment with 10^−5^ M malathion weakly induced ER transactivity	Can act as a potential agonist, activating the ER signaling pathway.

Unless otherwise stated, all findings listed were statistically significant (*p* < 0.05). MSI, microsatellite instability; LOH, loss of heterozygosity; ER, estrogen receptor.
